# Modulation of NRF-2 Pathway Contributes to the Therapeutic Effects of *Boswellia serrata* Gum Resin Extract in a Model of Experimental Autoimmune Myocarditis

**DOI:** 10.3390/antiox11112129

**Published:** 2022-10-28

**Authors:** Ramona D’Amico, Roberta Fusco, Marika Cordaro, Livia Interdonato, Rosalia Crupi, Enrico Gugliandolo, Davide Di Paola, Alessio Filippo Peritore, Rosalba Siracusa, Daniela Impellizzeri, Salvatore Cuzzocrea, Rosanna Di Paola

**Affiliations:** 1 Department of Chemical, Biological, Pharmaceutical and Environmental Sciences, University of Messina, Via F. Stagno D’Alcontres, 98166 Messina, Italy; 2 Department of Biomedical, Dental and Morphological and Functional Imaging, University of Messina, Consolare Valeria, 98166 Messina, Italy; 3 Department of Veterinary Sciences, Annunziata Campus, University of Messina, Viale Annunziata, 98168 Messina, Italy; 4 Department of Pharmacological and Physiological Science, Saint Louis University School of Medicine, Saint Louis, MO 63103, USA

**Keywords:** *Boswellia* gum extract, inflammation, NRF-2, immunity, cytokines

## Abstract

Myocarditis is a clinically dangerous disease that can result in death. Oxidative stress as well as inflammatory and immune responses play important roles in the development of myocarditis. Presently, more research has been carried out on anti-inflammatory treatment using natural compounds. The aim was to evaluate the anti-inflammatory and antioxidant effect of *Boswellia* gum resin extract in an experimental autoimmune myocarditis (EAM) and the involvement of molecular pathways. Rats were immunized with porcine cardiac myosin to ascertain EAM. The EAM rats were treated orally with *Boswellia* extract or vehicle for 21 days. EAM caused macroscopic and microscopic alterations with necrosis, inflammatory cell infiltration, fibrosis of the heart tissues, as well as clinical biochemical changes, cytokines release, altered immune response, and oxidative stress. Oral treatment with *Boswellia* markedly reduced myocardial damage, decreased inflammatory infiltrate, fibrosis, biochemical markers, such as lactate dehydrogenase and the creatine kinase, and heart weight/body weight ratio. In addition, low nitric oxide and malondialdehyde levels together with the upregulation of antioxidant nuclear factor erythroid 2–related factor 2 NRF-2 pathway were observed in EAM rats treated with *Boswellia*. Thus, *Boswellia* could be considered as a new natural extract to combat heart pathologies, such as autoimmune myocarditis.

## 1. Introduction

Heart disease is one of the world’s significant health problems, affecting both developed and developing countries. Acute myocarditis is a potentially fatal condition that frequently precedes the expansion of distended cardiomyopathy in humans [1]. Myocarditis is characterized by cardiac inflammation, oedema, cellular infiltration, apoptosis, and necrosis of cardiomyocytes [1]. A discrepancy of reactive oxygen species (ROS) and/or an unsatisfactory cellular antioxidant defense mechanism may play a decisive role in myocardial damage in acute myocarditis [2]. Increased ROS can cause serious cardiovascular dysfunction by attacking contractile molecules or ion channels directly. Furthermore, an imbalance in intracellular oxido-reductive state (redox) may activate stress-sensitive signaling pathways, boosting apoptosis and potentially contributing to the development of heart failure [3]. Cardiac myosin-induced myocarditis is a rat model of experimental autoimmune myocarditis (EAM) used to study autoimmunological pathways in inflammatory heart disorders. During the disease’s progression, myosin causes a localized inflammation by neutrophil and macrophage infiltration, which then activates mononuclear effector cells and initiates the EAM [1]. This EAM model is histologically similar to acute human myocarditis, with macrophage, lymphocyte, and neutrophil recruitment accompanying cardiac fibrosis [4]. Given that the present therapy choices for myocarditis are confined to symptoms as arrhythmias and heart failure, and that no other strategy has yet been discovered [5], innovative effectual therapeutic alternative is indispensable. Natural products are becoming more popular in the treatment of cardiovascular disease due to their security, less side effects, and reduced prices. In current years, preclinical and clinical research has concentrated on discovery novel phyto-medicines, such as plant extracts with significant anti-inflammatory and antioxidant action, and, in particular, active constituents for cardioprotection [5]. Some plants, such as the Indian plant *Boswellia serrata (B. serrata*), have antioxidant qualities that are useful in the treatment and prevention of various ailments. It belongs to the angiosperm family Burseraceae, which comprises phenolic compounds with a benzene ring, a carboxyl terminal, and one or more hydroxyl groups and/or methoxyl in the molecule [6]. It confers antioxidant properties on the system. *B. serrata* trunk extract has been used in traditional medicine in India and other eastern nations to treat inflammatory disorders such as arthritis, osteoarthritis, and IBD [7]. It is also useful in reducing lipid oxidation [7]. The active ingredient in *B. serrata* extract is known as boswellic acid (BA), which is composed of pentacyclic triterpenes with antioxidant qualities, such as anti-inflammatory, anti-atherosclerotic, anti-hepatotoxic, and anti-hyperlipidemic capabilities [8]. This acts as a free radical scavenger and sometimes as a metal chelator, acting in the propagation of the oxidative process [9]. The six main acids recognized are α and β boswellic acids (BA), acetylated α and β-boswellic acids (ABA), 11β-keto—boswellic acid (KBA), and 3-*O*-acetyl-11-keto-β boswellic acid (AKBA), which are responsible for inhibiting the inflammatory enzymes [10]. The major natural compounds present in in *B. serrata* extract include various classes. These are phenolic compounds, such as [2,7,8 trimethoxy-3 methyl-5,6- methylenedioxynaphtho-1,4-quinine], [diterpenoids (1S, 2E, 4S, 6E, 8R, 11S, 12R)-8, 11-Epoxy-2, 6-thunbergadiene-4, 12-diol], esters of acetic acid, such as [(E,E,10S)-10,11-epoxy-3,7,11-trimethyl-2,6-dodecadiene-1-yl] acetate, a quinoline alkaloid; benzo(h) quinolino (1″,2″:1′,2′) imidazo (4′,5′:4,5) imidazo (1,2-a) benzo-(h) quinoline, and ketones as (3E)-4- (5,5- dimethyl-1- oxaspiro [2,5] oct-4-yl)-3-buten-2-one] [11]. Thus, the aim of this study was to evaluate the anti-inflammatory and antioxidants effects of *Boswellia serrata* gum resin extract in a rat model of autoimmune myocarditis and the molecular mechanisms involved.

## 2. Materials and Methods

### 2.1. Materials

The powder of *Boswellia serrata* gum resin extract (from *Burseraceae* family) (lot. S2111560) was purchased (Fontana standardized natural active principles, Canosa di Puglia, BT Italy). The extract was arranged as a fine homogenized suspension (vehicle, 2% gum acacia) for oral administration. All compounds used were bought from Sigma-Aldrich Company Ltd. (Milan, Italy).

### 2.2. Animals

Sprague Dawley SD rats (male, 200–220 g) were acquired from Envigo Milan, Italy, and kept in the animal home under controlled conditions. The current study was authorized by Messina University’s Animal Welfare Evaluation Board. All animal research was conducted with new Italian legislation (D.Lgs 2014-26), as well as EU rules (EU Directive 2010-63). Approval code n° 89/2021-PR.

### 2.3. Carrageenan (CAR)-Induced Paw Edema (Preliminary Data)

Paw edema was implemented as previously indicated by a subplantar inoculation of CAR [12].

### 2.4. Experimental Groups (Preliminary Data)

Initially, we tested the dose–response of *Boswellia serrata* gum resin extract in a model of CAR-induced paw edema. Rats were distributed into distinctive groups:

- CAR + vehicle: rats were injected with CAR and administered orally with vehicle.
- CAR *+* *Boswellia* extract (10 mg/kg): rats were injected with CAR and *Boswellia* extract was administered orally 30 min before CAR injection.
- CAR *+ Boswellia* extract (50 mg/kg): rats were injected with CAR and *Boswellia* extract was administered orally 30 min before CAR injection.
- CAR *+* *Boswellia* extract (100 mg/kg): rats were injected with CAR and *Boswellia* extract was administered orally 30 min before CAR injection.
- Sham-operated groups received saline and were treated orally with vehicle or *Boswellia* (data not shown).

### 2.5. Induction of Experimental Autoimmune Myocarditis

Purified pig cardiac myosin (Sigma Chemical Co., St. Louis, MO, USA) was emulsified with an equal amount of complete Freund’s adjuvant (CFA, Difco, Sparks, MD, USA) supplemented with mycobacterium tuberculosis H37RA (10 mg/mL, Difco). Subcutaneously, 0.2 cc of emulsion was injected into the footpads of rats. Following immunization, the rats were given extract or vehicle orally for 21 days [13].

### 2.6. Experimental Groups

The animals were indiscriminately distributed into the following groups, n = 12 for each

(1) Sham + vehicle: rats were injected with CFA and treated orally with vehicle every day for 21 days.
(2) Sham + *Boswellia* gum extract: rats received CFA and were treated orally with *Boswellia* gum extract 100 mg/kg every day for 21 days.
(3) EAM + vehicle: rats were injected with emulsified porcine myosin in CFA and treated orally with vehicle every day for 21 days.
(4) EAM + *Boswellia* gum extract: rats were injected with emulsified porcine myosin in CFA and treated orally with *Boswellia* gum extract 100 mg/kg every day for 21 days. At 3 weeks post induction, (21 day), rats were killed, blood and heart tissues were collected. The route and dose administration of *Boswellia* gum extract were selected based on [14]. Since no significant difference was discovered between the sham + vehicle and sham + *Boswellia* gum extract, simply the data of sham + vehicle groups were displayed.

### 2.7. Heart Rate and Blood Pressure Measurements

At 3 weeks post immunization, under isoflurane-induced anaesthesia, catheters were positioned in the right carotid artery. Systemic pressure monitoring was recorded by ADistrument BP Blood Pressure Transducer for (MLT0699) displayed on personal computer. All data concerning mean blood pressure and heart rate (HR) were collected and analyzed using t PowerLab data acquisition system (AD Instruments) and LabChart version 7.2 software.

### 2.8. Biochemical Parameters

The lactate dehydrogenase and the creatine kinase (CK-MB) activities in the rat serum were estimated using offered LDH and CK assay kits (Sigma–Aldrich, Milan, Italy).

### 2.9. Heart Weight, hw/Body Weight, bw (Hw/Bw)

The rat’s body weight was measured during the experiment. At 21 days post immunization, the rats were sacrificed, and hearts were isolated to calculate relative Hw/Bw.

### 2.10. Cytokines Measurements

The concentration of serum cytokines TNF-α, IL-17, IL-10, and IL-6 was determined using ELISA kits (R&D Systems; Minneapolis, MN, USA). Serum IL-2 and IL-4 were determined by assay kits (Eagle Biosciences, Inc., Amherst, NH, USA) [15,16,17].

### 2.11. Macroscopic Evaluation

Heart macroscopic observations were divided into four grades based on criteria as indicated [18].

### 2.12. Histological Analysis

The excised myocardium was kept in formalin and the sections were then embedded in paraffin [19,20,21,22]. The heart tissue was sectioned and stained with hematoxylin and eosin. The sections were scored for myocarditis as follows in blinded (score 0–4) [13]. Moreover, the area of inflammatory cells was evaluated using image J (National Institutes of Health, Bethesda, MD, USA), which was shown as the ratio of area of inflammatory cells to that of total area [23]. Paraffin-embedded heart tissues were also stained with Masson’s trichrome according to the manufacturer’s protocol (Bio-Optica, Milan, Italy).

### 2.13. Immunohistochemistry for Smooth Muscle Alpha-Actin (α-Sma) and Transforming Growth Factor Beta (TGF-β) and Nuclear Factor Erythroid 2–Related Factor 2 (NRF-2)

Immunohistochemical analysis was performed as previously described [19,20,24,25,26,27]. The sections were incubated overnight with primary antibodies: anti- α-sma antibody (1:100, Santa Cruz Biotechnology (SCB), anti-TGF-β antibody (1:200, Millipore) or anti-NRF-2 (1:100, SCB). Sections were cleansed with PBS, then treated as indicated previously [24], and blindly scored using a Leica DM6 microscope (Leica Microsystems SpA, Milan, Italy) by a standard process [28,29].

### 2.14. Malondialdehyde (MDA) Levels

The lipid peroxidation in the cardiac homogenate was determined, as previously described [19,30,31,32].

### 2.15. Determination of Nitric Oxide (NO)

The level of NO in the cardiac homogenate was measured by assaying total nitrate/nitrite, the stable products of NO oxidation, as described [30,33].

### 2.16. Immunofluorescence for CD4, CD8, CD45, CD11β

The following primary antibodies were used: anti-CD4 rabbit (Abcam; 1:50 in PBS, *v*/*v*), or anti-CD8 (Abcam, rabbit 1:50), anti CD45 rabbit (Abcam; 1:50 in PBS, *v*/*v*), or anti-CD11β mouse (Abcam 1:50 in PBS, *v*/*v*), as previously described [34,35,36,37,38].

### 2.17. Western Blot for NRF-2, HO-1 and MnSOD

Cytosolic and nuclear extracts were prepared as previously described [39,40,41,42,43,44,45]. The following primary antibodies were used: anti-NRF-2 61kDa (sc-365949, 1:1000, SCB), anti-HO-1 32 kDa (sc-136960, 1:1000 SCB), anti-MnSOD 23 kDa (1:500; Millipore) in 1 × PBS, 5% (*w*/*v*) non-fat dried milk, 0.1% Tween-20 at 4 °C overnight. Anti-β-actin or anti-lamin A/C antibodies were used as controls. The procedure of expression of protein bands was previously described [39]. 

### 2.18. Statistical Evaluation

All results are given as the mean standard error (SEM) of N observations, where N = number of rats. For histology/immunohistochemistry, the images are the result of three distinct experiments. *p* value < 0.05 was significant. For macroscopic and pathological scores data, the Kruskal–Wallis test was used with Dunn’s post hoc test for multiple comparisons. For all other data, one-way ANOVA was employed, with a Bonferroni post-hoc test.

## 3. Results

### 3.1. Effects of Boswellia Gum Extract on Paw Damage: Preliminary Data

**Figure 1:**
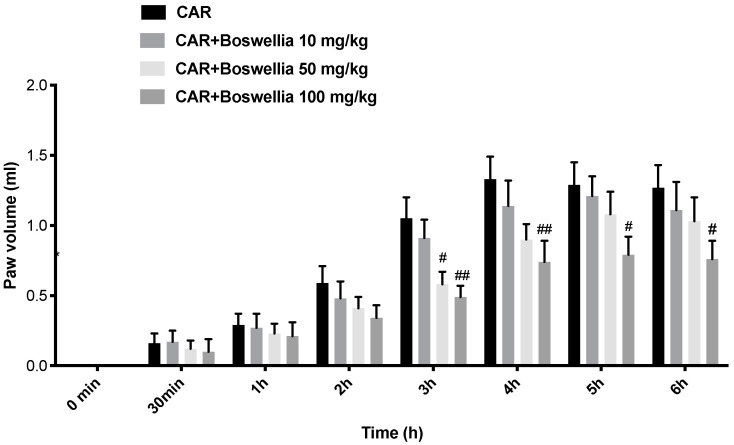
Preliminary data of anti-inflammatory effects of *Boswellia* extract at different doses 10, 50, and 100 mg/kg. Values are means ± SEM of 6 animals for each group; ## *p* < 0.01 vs. CAR. # *p* < 0.05 vs. CAR.

To well comprehend which dose of *Boswellia* extract could be effective, we preliminarily studied the effects of *Boswellia* extract on paw edema. We established three doses: 10, 50, and 100 mg/kg based on the literature [46]. CAR injection caused a time-dependent rise in the paw volume compared to the shams. *Boswellia* extract (10 mg/kg) was not able to lessen the paw edema, while the higher doses of 50 (at time of 3 h) and in particular at 100 mg/kg were able to lessen the paw swelling from 3 to 6 h post CAR (Figure 1). 

### 3.2. Effects of Boswellia Gum Extract on Mean Blood Pressure, Heart Rate, Biochemical Parameters, and Hw/Bw in EAM Rats

**Figure 2:**
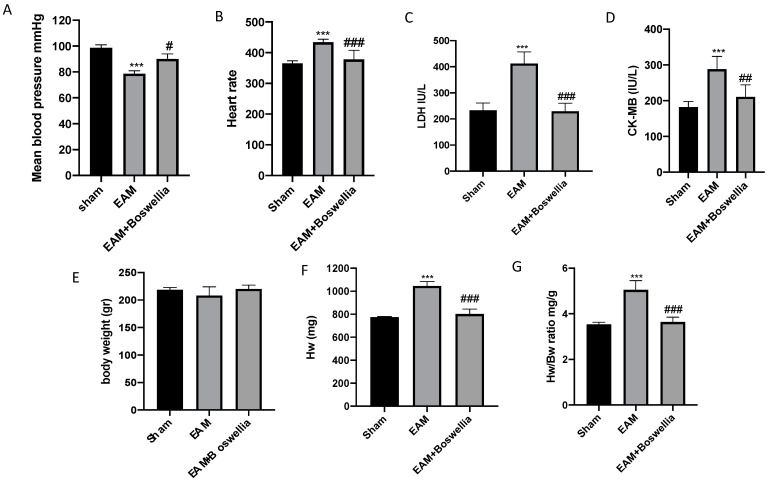
Effect of *Boswellia* extract on mean blood pressure, heart rate, Hw/Bw and biochemical parameters. Mean blood pressure and heart rate (**A**,**B**), serum levels of LDH (**C**), CK-MB (**D**), Body weight, Hw and Hw/Bw (**E**–**G**) measures at last day of experiment. *Boswellia* extract administration was able to ameliorate all these parameters. Values = means ± SEM of 6 animals for each set; *** *p* < 0.001 vs. sham; ^###^ *p* < 0.001 vs. EAM ^##^ *p* < 0.01 vs. EAM ^#^ *p* < 0.05 vs. EAM.

At 21 days post immunization, the EAM + vehicle group showed significantly fast heart rate and failure of mean blood pressure (Figure 2A,B). Oral administration of *Boswellia* statistically suppressed the heart rate acceleration and decline of mean blood pressure (Figure 2A,B). In addition, biomarkers of cardiac function alterations were also evaluated. Increased serum levels CK-MB and LDH were perceived in EAM compared to sham rats, while oral *Boswellia* caused a significant decrease in serum levels of both CK-MB and LDH (Figure 2C,D). In addition, HW corrected by BW was significantly higher in the EAM group with hearts markedly enlarged, but this increase was prevented by *Boswellia* treatment, whereas the sham group showed no changes (Figure 2E–G). Thus, the oral administration of *Boswellia* significantly diminished the heart weight/body weight ratio compared to vehicle groups (Figure 2E–G).

### 3.3. Effects of Boswellia Gum Extract on Macroscopic and Microscopic Damage and Fibrosis in EAM Rats

**Figure 3:**
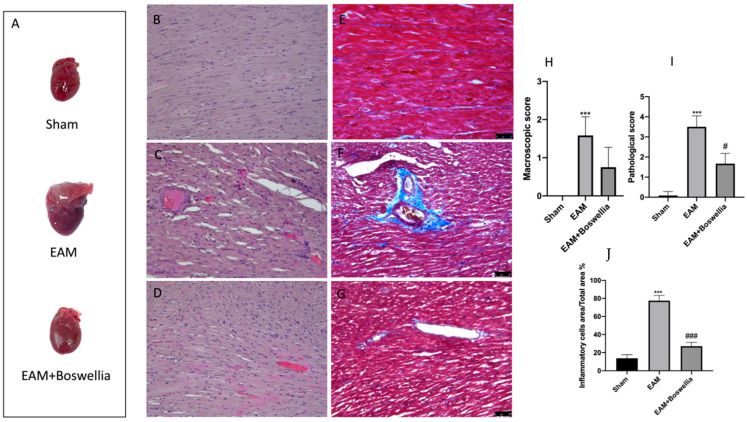
Effect of *Boswellia* extract on macroscopic and microscopic scores. Macroscopic panel (**A**), Histological and Masson stainings were evaluated in heart tissues of sham (**B**,**E**), EAM + vehicle (**C**,**F**) EAM + *Boswellia* gum extract (**D**,**G**). Macroscopic and microscopic scores were shown (**H**,**I**). Percentage of Inflammatory cells area/total area (**J**). *Boswellia* extract administration was able to reduce macroscopic and microscopic scores and inflammatory infiltrate. Values = means ± SEM of 6 animals for each set. *** *p* < 0.001 vs. sham; ^###^ *p* < 0.001 vs. EAM. ^#^ *p* < 0.05 vs. EAM.

Macroscopic and microscopic evaluations of the heart were evaluated at three weeks post immunization. No macroscopic change and preserved myocardial structure were found in sham groups (Figure 3A,B,H–J). Significant discolored areas were observed in the hearts of EAM vehicle but less in EAM + *Boswellia* (Figure 3A,H). E-stained sections of the EAM group of rats confirmed the presence of severe myocarditis characterized by much inflammatory cells infiltration and myocardial necrosis with respect to controls (Figure 3C,I,J). However, *Boswellia* treatment especially improved cardiac structure after the three-week treatment and reduced the presence of inflammatory infiltrate (Figure 3D,I,J). In addition, myocardial fibrosis was also observed in EAM heart tissues compared to controls (Figure 3E,F). *Boswellia* oral administration was able to reduce fibrotic process (Figure 3G).

### 3.4. Effects of Boswellia Gum Extract on α-sma and TGF-β in EAM Rats

**Figure 4:**
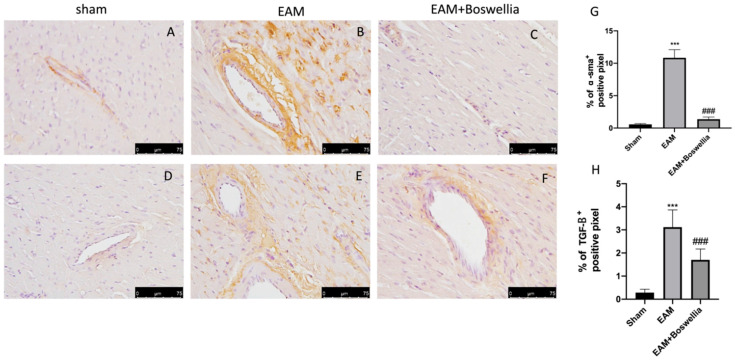
Effect of *Boswellia* extract on α-sma and TGF-β expressions. (**A**,**D**) shams, (**B**,**E**), EAM + vehicle, (**C**,**F**), EAM + *Boswellia* gum extract. *Boswellia* extract administration was able to reduce α-sma and TGF-β expressions. The results are expressed as % of positive pixels (**G**,**H**). Values = means ± SEM of 6 animals for each set; *** *p* < 0.001 vs. sham, ^###^ *p* < 0.001 vs. EAM. Scale bar: 75 μm. Magnification (40×).

The fibrotic process was also confirmed by immunohistochemistry for α-sma and TGF-β. Increased expression of α-sma and TGF-β was found in EAM rats + vehicle compared to sham groups (Figure 4A,B,D,E,G,H). The oral administration of *Boswellia* reduced in a significant way the immunostaining for α-sma and TGF-β (Figure 4C,F,G,H).

### 3.5. Effects of Boswellia Gum Extract on Cytokines Release in EAM Rats

**Figure 5:**
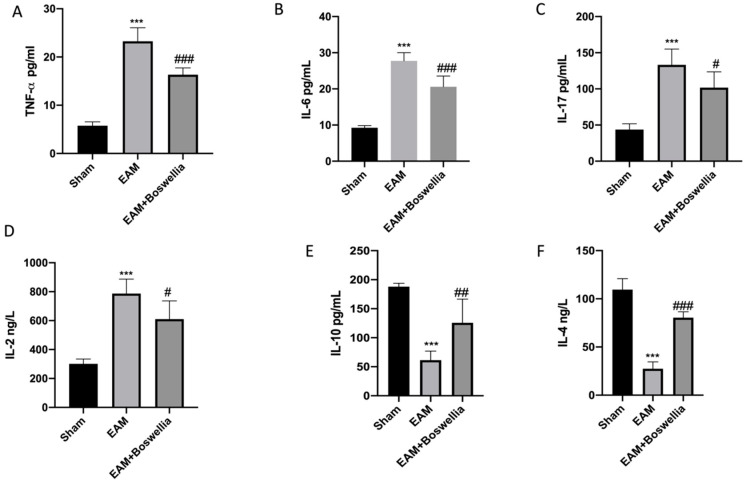
Effect of *Boswellia* extract on cytokines levels. Serum levels of TNF-α (**A**), IL-6 (**B**), IL-17 (**C**), IL-2 (**D**), IL-10 (**E**), IL-4 (**F**). *Boswellia* extract administration was able to reduce proinflammatory cytokines and increase anti-inflammatory cytokines. Values = means ± SEM of 6 animals for each set. *** *p* < 0.001 vs. sham; ^##^ *p* < 0.01 vs. EAM. ^###^ *p* < 0.001 vs.EAM. ^#^ *p* < 0.05 vs. EAM.

EAM induction caused a marked increase of proinflammatory cytokine levels, such as TNF-α, IL-6, IL-17, and IL-2, compared to sham (Figure 5A–D). Oral administration of *Boswellia* extract reduced these proinflammatory cytokine levels (Figure 5A–D). In addition, in EAM rats, an important reduction of anti-inflammatory cytokines levels was observed, such as IL-4 and IL-10 (Figure 5E,F). *Boswellia* was able to increase the levels of IL-4 and IL-10 (Figure 5E,F).

### 3.6. Effects of Boswellia Gum Extract on CD4, CD8, CD45 and CD11β Expression in EAM Rats

**Figure 6:**
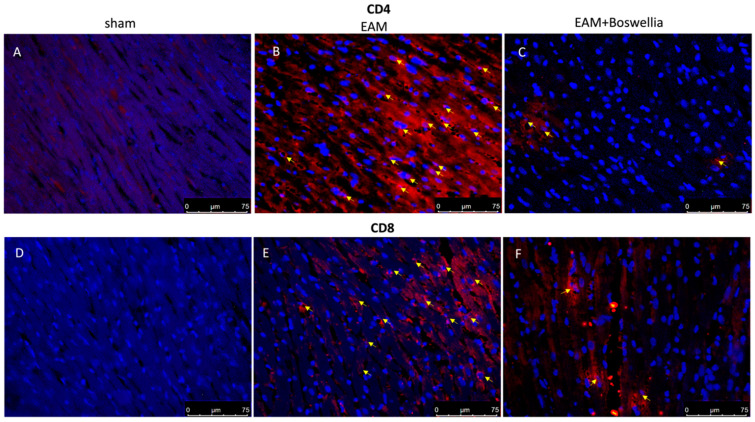
Effect of *Boswellia* extract on CD4 and CD8 expressions. Immunofluorescence for CD4 and CD8 (red) (see yellow arrows) in heart sections of sham animals (**A**,**D**), EAM + vehicle (**B**,**E**) and EAM + *Boswellia* gum extract (**C**,**F**). *Boswellia* extract administration was able to reduce CD4 and CD8 expressions.. Scale bar: 75 μm. Magnification (40×).

**Figure 7:**
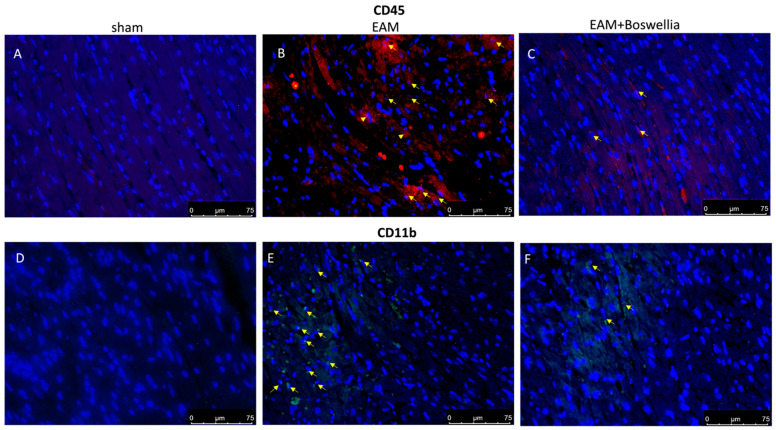
Effect of *Boswellia* extract on CD45 and CD11β expressions. Immunofluorescence for CD45 “red” and CD11β “green” (see yellow arrows) in heart sections of sham (**A**,**D**), EAM + vehicle (**B**,**E**) and EAM + *Boswellia* gum extract (**C**,**F**). *Boswellia* extract administration was able to reduce CD45 and CD11β expressions. Scale bar: 75 μm. Magnification (40×).

Cardiac myosin injection caused an important inflammatory cell infiltration. Immunohistochemistry on day 21 revealed the increased expression of CD4, CD8, CD45 (markers for immune cells) and CD11β (marker for macrophages) observed in the EAM + vehicle group compared to controls (Figure 6 and Figure 7A,B,D,E). Oral treatment with *Boswellia* reduced this immunopositivity in a significant way for all markers (Figure 6 and Figure 7C,F).

### 3.7. Effects of Boswellia Gum Exctract on Oxidative Stress in EAM Rats

**Figure 8:**
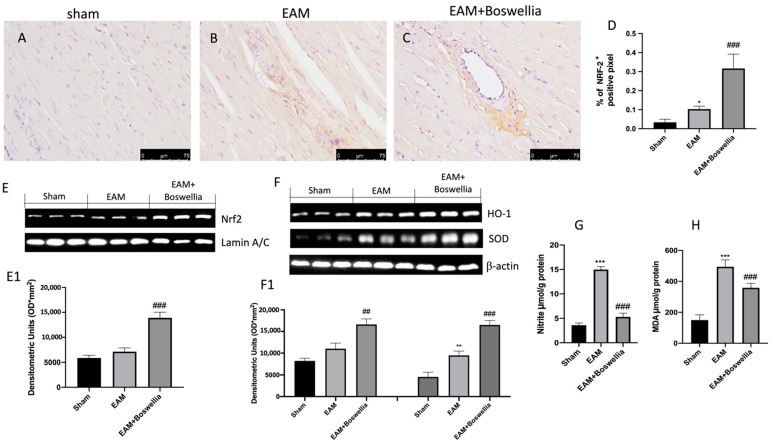
Effect of *Boswellia* extract on NRF-2 pathway and evaluation of NO release and MDA levels. NRF-2 immunohistochemistry for (**A**) sham group, (**B**), EAM + vehicle group, (**C**) EAM + *Boswellia* gum extract. The result is expressed as % of positive pixels (**D**). Scale bar: 75 μm. Magnification (40×). Western blot for NRF-2 (**E**,**E1**), HO-1 and SOD (**F**,**F1**). Exposed is a characteristic blot of lysates from 6 animals/set, together with a densitometric evaluation for all (**E1**,**F1**). Nitrite levels (**G**), MDA levels (**H**). *Boswellia* extract administration was able to activate NRF-2 pathway as well as reduce nitrite and MDA levels. Values = means ± SEM of 6 animals for each set. *** *p* < 0.001 vs. sham. ** *p* < 0.01 vs. sham. * *p* < 0.05 vs. sham ^##^ *p* < 0.01 vs. EAM. ^###^ *p* < 0.001 vs. EAM.

To evaluate the effects of *Boswellia* on oxidative stress and to assess whether it could act by promoting antioxidant pathway NRF-2, we performed immunohistochemistry and Western blot analyses in myocardial tissues. The expression of NRF-2 was physiologically increased in the EAM vehicle group (Figure 8B,D) compared to controls (Figure 8A,D), while *Boswellia* significantly augmented NRF-2 immuno-reactivity (Figure 8C,D). By Western blot, a small rise of Nrf-2 was perceived in EAM rats with respect to sham (Figure 8E,E1). *Boswellia* meaningfully upregulated Nrf-2 compared to the vehicle (Figure 8E,E1). Western blot showed that *Boswellia* meaningfully augmented HO-1 and SOD protein expression compared to vehicle (Figure 8F,F1).

In addition, nitrite/nitrate (to detect the release of NO) and MDA level measurements (to detect lipid peroxidation) were also evaluated. Myocarditis induction caused increased MDA and nitrite levels compared to sham (Figure 8G,H). *Boswellia* treatment was able to significantly reduce nitrite and MDA levels (Figure 8G,H).

## 4. Discussion

Myocarditis is an inflammatory illness of the myocardium that has been connected to the development of autoimmunity. It is usually associated with cardiotropic infections. A murine model of EAM called myosin-induced myocarditis in rats is utilized to study the pathogenesis of acute and chronic heart failure and DCM [47]. Pathologically, myocarditis is designated as mono-nuclear or mixed cell infiltration with necrosis of myocytes in the presence/absence of fibrosis [48]. Excessive ROS generation and subsequent oxidative stress are known to cause the release of inflammatory cytokines and chemokines involved in leukocyte migration to cardiac tissue [5]. Previous research has revealed that there are various potential targets in myocarditis [49], however, there is no particular and effective treatment for myocarditis. Thus, novel therapeutic techniques aimed at alleviating myocarditis are required. Several medicinal herbs with antioxidant characteristics can also provide cardioprotection. For example, recently, another natural extract, *Melissa officinalis* extracts (MOEs), was shown ameliorate cardiac function, structure, and morphology, thereby improving the antioxidant defense system [5]. *Boswellia* species are candidates for the same potential cardioprotective activity, in particular the extract of *B. serrata*, which has also shown free radical scavenging action [50]. *Boswellia serrata* has been employed in Ayurvedic medicine since ancient India. Exudates of *B. serrata* stem bark or its main elements, boswellic acids, have anti-inflammatory [51,52,53], anti-cancer [54], and anti-ulcerous properties [55]. Toxicology animal studies on *B. serrata* resin revealed no significant histopathological, genotoxic, or hematological alterations after use of this resin [56,57]. Furthermore, the side effects in humans are minor, and some consumers have complained of nausea, acid reflux, and digestive issues [56]. 

Based on this, we evaluated the antioxidant effects of *Boswellia serrata* gum resin extract in a rat model of experimental myocarditis. In our work, the induction of EAM was validated histopathologically by significant inflammatory infiltration and fibrosis of the cardiac tissues. The cardio-toxic effect of myocarditis was also established by elevated serum CK-MB and LDH activity levels together with low mean blood pressure and increase of heart rate in the EAM- rats. Surprisingly, a three-week therapy with *Boswellia* gum extract enhanced cardiac architecture by decreasing inflammatory infiltration and myocarditis-induced fibrosis. Furthermore, in the EAM *Boswellia*-treated group, all biochemical markers, including Hw/Bw, macroscopic and microscopic scores, were less than in the EAM vehicle-treated group.

The inflammatory response is crucial in the pathogenesis of cardiovascular disease. At the start of EAM, the injected myosin targets myocardial tissue, activating cardiac-resident immune cells or circulating cells that have moved to the heart [58]. A substantial body of evidence demonstrates that T helper 1 (Th1) cytokines (such as IL-2 and TNF) have a role in the pathogenesis of EAM [59]. A variety of immune cells, including CD4, CD8, and granulocytes, have been linked to localized myocardial damage, which can result in cardiomyocyte mortality and cardiac contractile failure [60,61]. CD4 cells play a vigorous role in the cardiac autoimmunity in myocarditis [62] and CD8 cells have been shown to contribute to myocarditis gravity [60]. At the crucial stage of the disease, CD11β+ represent the majority of heart-infiltrating mononuclear cells [63]. Our study revealed that the EAM induced group showed increased proinflammatory cytokines and reduced levels of anti-inflammatory cytokines with enhanced expression of positive T cells (CD4, CD8, CD45 and CD11β), while treatment with *Boswellia* gum extract was able to increase IL-10 and IL-4 levels, reduce TNF-α, IL-6, IL-2, and IL-17, and to suppress the expression of CD4, CD8, CD45, and CD11β. This report is in agreement with previous studies in which *Boswellia serrata* inhibited IL-1β, TNF-α, interferon-γ (IFN-γ), and enhanced the production of IL-10 [64]. 

There is mounting evidence that both free radicals and oxidative stress play a vital role in the development of heart failure [65]. Several studies have found that boswellic acids and *B. serrata* extract can combat free radicals that induce inflammation, preventing tissue damage and fibrosis. In particular, it has been demonstrated that *B. serrata* therapy reduced oxidative stress, increased total antioxidant capacity, and decreased the expression of TNF, TGF-β, and IL-6 [66] as well as displayed significantly reduced lipid peroxidation, nitric oxide and inducible nitric oxide synthase iNOS levels, and presented enhancements in tissue damage in animals with ulcerative colitis [8]. Activation of the Nrf2-ARE-driven gene regulatory pathway by a variety of natural compounds confers protection against various diseases of oxidative stress origin. This pathway is considered a promising therapeutic target for natural phytochemicals derived from various natural products [5]. Several studies reported that *Boswellia* was able to have beneficial effects by the modulation of NRF-2/HO-1 pathway [67,68]. A previous report found that boswellic acids protect against doxorubicin-induced hepatotoxicity through influencing the Nrf2/HO-1 Defense Pathway [67]. In particular, in the case of isoproterenol-induced myocardial injury in rats, it was reported that acetyl-11-keto-β-boswellic acid in combination exercised antioxidant action possibly by targeting NRF-2 [69]. In this study, we reported that EAM rats showed increased NO release and lipid peroxidation together with reduced antioxidant NRF-2 expression. *Boswellia* gum extract administration was able to reduce NO release and lipid peroxidation and to increase NRF-2 expression. Among the various antioxidant enzymes, an important role is played by the superoxide dismutase (SOD) [70]. The effect of *Boswellia* gum extract on the NRF-2/HO-1 pathway was also confirmed by Western blot analysis showing that the expression of NRF-2, HO-1, and SOD was significantly increased.

## 5. Conclusions

**Figure 9:**
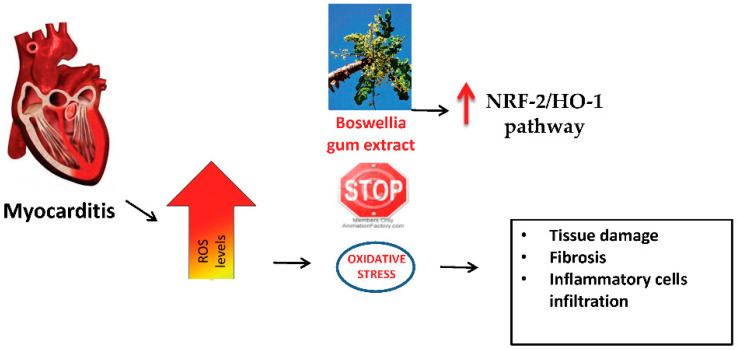
Summary of the effects of *Boswellia* gum extract during EAM.

In conclusion, *Boswellia* gum extract administration reduced the severity of myocarditis, modulating inflammatory and immune responses and increasing antioxidant defense by the upregulation of the NRF-2/HO-1 pathway. Thus, it could be considered as a new natural extract to combat heart pathologies, such as autoimmune myocarditis (Figure 9).

However, this study presents a limitation due to a lack of phytochemical characterization of the *Boswellia* extract which would have provided additional information on which components are involved in the antioxidant response. Thus, future studies are needed.

## Data Availability

All of the data is contained within the article.

## References

[1] Ahmed A.M., El Fouhil A.F., Mohamed R.A., Atteya M., Abdel-Baky N.A., AlRoalle A.H., Aldahmash A.M. (2015). Curcumin ameliorates experimental autoimmune acute myocarditis in rats as evidenced by decrease in thioredoxin immunoreactivity. Folia Morphol..

[2] Yuan Z., Kishimoto C., Shioji K., Nakamura H., Yodoi J., Sasayama S. (2003). Temocapril treatment ameliorates autoimmune myocarditis associated with enhanced cardiomyocyte thioredoxin expression. Mol. Cell. Biochem..

[3] Wongcharoen W., Jai-Aue S., Phrommintikul A., Nawarawong W., Woragidpoonpol S., Tepsuwan T., Sukonthasarn A., Apaijai N., Chattipakorn N. (2012). Effects of Curcuminoids on Frequency of Acute Myocardial Infarction after Coronary Artery Bypass Grafting. Am. J. Cardiol..

[4] Liu W., Nakamura H., Shioji K., Tanito M., Oka S.-I., Ahsan M.K., Son A., Ishii Y., Kishimoto C., Yodoi J. (2004). Thioredoxin-1 Ameliorates Myosin-Induced Autoimmune Myocarditis by Suppressing Chemokine Expressions and Leukocyte Chemotaxis in Mice. Circulation.

[5] Draginic N.D., Jakovljevic V.L., Jeremic J.N., Srejovic I.M., Andjic M.M., Rankovic M.R., Sretenovic J.Z., Zivkovic V.I., Ljujic B.T., Mitrovic S.L. (2022). Melissa officinalis L. Supplementation Provides Cardioprotection in a Rat Model of Experimental Autoimmune Myocarditis. Oxidative Med. Cell. Longev..

[6] Rahimi R. (2010). A review of the efficacy of traditional Iranian medicine for inflammatory bowel disease. World J. Gastroenterol..

[7] Kimmatkar N., Thawani V., Hingorani L., Khiyani R. (2003). Efficacy and tolerability of Boswellia serrata extract in treatment of osteoarthritis of knee—A randomized double blind placebo controlled trial. Phytomedicine.

[8] Hartmann R.M., Martins M.I.M., Tieppo J., Fillmann H.S., Marroni N.P. (2012). Effect of Boswellia serrata on Antioxidant Status in an Experimental Model of Colitis Rats Induced by Acetic Acid. Am. J. Dig. Dis..

[9] Krüger P., Daneshfar R., Eckert G.P., Klein J., Volmer D.A., Bahr U., Müller W.E., Karas M., Schubert-Zsilavecz M., Abdel-Tawab M. (2008). Metabolism of Boswellic Acids In Vitro and In Vivo. Drug Metab. Dispos..

[10] Roy N.K., Parama D., Banik K., Bordoloi D., Devi A.K., Thakur K.K., Padmavathi G., Shakibaei M., Fan L., Sethi G. (2019). An Update on Pharmacological Potential of Boswellic Acids against Chronic Diseases. Int. J. Mol. Sci..

[11] Gomaa A.A., Makboul R.M., Al-Mokhtar M.A., Nicola M.A. (2019). Polyphenol-rich Boswellia serrata gum prevents cognitive impairment and insulin resistance of diabetic rats through inhibition of GSK3β activity, oxidative stress and pro-inflammatory cytokines. Biomed. Pharmacother..

[12] Britti D., Crupi R., Impellizzeri D., Gugliandolo E., Fusco R., Schievano C., Morittu V.M., Evangelista M., Di Paola R., Cuzzocrea S. (2017). A novel composite formulation of palmitoylethanolamide and quercetin decreases inflammation and relieves pain in inflammatory and osteoarthritic pain models. BMC Vet. Res..

[13] Zhang Q., Hu L.-Q., Li H.-Q., Wu J., Bian N.-N., Yan G. (2019). Beneficial effects of andrographolide in a rat model of autoimmune myocarditis and its effects on PI3K/Akt pathway. Korean J. Physiol. Pharmacol..

[14] Umar S., Umar K., Sarwar A.H., Khan A., Ahmad N., Ahmad S., Katiyar C.K., Husain S.A., Khan H.A. (2014). Boswellia serrata extract attenuates inflammatory mediators and oxidative stress in collagen induced arthritis. Phytomedicine.

[15] Di Paola D., Natale S., Gugliandolo E., Cordaro M., Crupi R., Siracusa R., D’Amico R., Fusco R., Impellizzeri D., Cuzzocrea S. (2022). Assessment of 2-Pentadecyl-2-oxazoline Role on Lipopolysaccharide-Induced Inflammation on Early Stage Development of Zebrafish (*Danio rerio*). Life.

[16] Lu Y.-F., Zhang Q.-Y., Wang L.-H., Liu X.-Y., Zhang S.-X. (2017). The protective effects of taurine on experimental autoimmune myocarditis. Eur. Rev. Med. Pharmacol. Sci..

[17] Petrosino S., Cordaro M., Verde R., Schiano Moriello A., Marcolongo G., Schievano C., Siracusa R., Piscitelli F., Peritore A.F., Crupi R. (2018). Oral Ultramicronized Palmitoylethanolamide: Plasma and Tissue Levels and Spinal Anti-hyperalgesic Effect. Front. Pharmacol..

[18] Milenković M., Arsenović-Ranin N., Stojić-Vukanić Z., Bufan B., Vučićević D., Jančić I. (2010). Quercetin Ameliorates Experimental Autoimmune Myocarditis in Rats. J. Pharm. Pharm. Sci..

[19] di Paola R., Cordaro M., Crupi R., Siracusa R., Campolo M., Bruschetta G., Fusco R., Pugliatti P., Esposito E., Cuzzocrea S. (2016). Protective Effects of Ultramicronized Palmitoylethanolamide (PEA-um) in Myocardial Ischaemia and Reperfusion Injury in VIVO. Shock.

[20] D’Amico R., Fusco R., Gugliandolo E., Cordaro M., Siracusa R., Impellizzeri D., Peritore A.F., Crupi R., Cuzzocrea S., Di Paola R. (2019). Effects of a new compound containing Palmitoylethanolamide and Baicalein in myocardial ischaemia/reperfusion injury in vivo. Phytomedicine.

[21] Di Paola D., Capparucci F., Abbate J.M., Cordaro M., Crupi R., Siracusa R., D’Amico R., Fusco R., Genovese T., Impellizzeri D. (2022). Environmental Risk Assessment of Oxaliplatin Exposure on Early Life Stages of Zebrafish (*Danio rerio*). Toxics.

[22] Fusco R., Cordaro M., Genovese T., Impellizzeri D., Siracusa R., Gugliandolo E., Peritore A., D’Amico R., Crupi R., Cuzzocrea S. (2020). Adelmidrol: A New Promising Antioxidant and Anti-Inflammatory Therapeutic Tool in Pulmonary Fibrosis. Antioxidants.

[23] Hirakawa H., Zempo H., Ogawa M., Watanabe R., Suzuki J.-I., Akazawa H., Komuro I., Isobe M. (2015). A DPP-4 Inhibitor Suppresses Fibrosis and Inflammation on Experimental Autoimmune Myocarditis in Mice. PLoS ONE.

[24] Cordaro M., Impellizzeri D., Gugliandolo E., Siracusa R., Crupi R., Esposito E., Cuzzocrea S. (2016). Adelmidrol, a Palmitoylethanolamide Analogue, as a New Pharmacological Treatment for the Management of Inflammatory Bowel Disease. Mol. Pharmacol..

[25] Fusco R., Siracusa R., D’Amico R., Cordaro M., Genovese T., Gugliandolo E., Peritore A., Crupi R., Di Paola R., Cuzzocrea S. (2020). Mucosa-Associated Lymphoid Tissue Lymphoma Translocation 1 Inhibitor as a Novel Therapeutic Tool for Lung Injury. Int. J. Mol. Sci..

[26] Fusco R., Gugliandolo E., Siracusa R., Scuto M., Cordaro M., D’Amico R., Evangelista M., Peli A., Peritore A., Impellizzeri D. (2020). Formyl Peptide Receptor 1 Signaling in Acute Inflammation and Neural Differentiation Induced by Traumatic Brain Injury. Biology.

[27] Peritore A.F., D’Amico R., Siracusa R., Cordaro M., Fusco R., Gugliandolo E., Genovese T., Crupi R., Di Paola R., Cuzzocrea S. (2021). Management of Acute Lung Injury: Palmitoylethanolamide as a New Approach. Int. J. Mol. Sci..

[28] Sawant S., Gokulan R., Dongre H., Vaidya M., Chaukar D., Prabhash K., Ingle A., Joshi S., Dange P., Joshi S. (2016). Prognostic role of Oct4, CD44 and c-Myc in radio–chemo-resistant oral cancer patients and their tumourigenic potential in immunodeficient mice. Clin. Oral Investig..

[29] Varghese F., Bukhari A.B., Malhotra R., De A. (2014). IHC Profiler: An Open Source Plugin for the Quantitative Evaluation and Automated Scoring of Immunohistochemistry Images of Human Tissue Samples. PLoS ONE.

[30] Abdel-Wahab B.A., Metwally M.E., El-Khawanki M.M., Hashim A.M. (2014). Protective effect of captopril against clozapine-induced myocarditis in rats: Role of oxidative stress, proinflammatory cytokines and DNA damage. Chem. Interact..

[31] Di Paola D., Iaria C., Capparucci F., Cordaro M., Crupi R., Siracusa R., D’Amico R., Fusco R., Impellizzeri D., Cuzzocrea S. (2021). Aflatoxin B1 Toxicity in Zebrafish Larva (*Danio rerio*): Protective Role of *Hericium erinaceus*. Toxins.

[32] Gugliandolo E., Peritore A.F., D’Amico R., Licata P., Crupi R. (2020). Evaluation of Neuroprotective Effects of Quercetin against Aflatoxin B1-Intoxicated Mice. Animals.

[33] Crupi R., Palma E., Siracusa R., Fusco R., Gugliandolo E., Cordaro M., Impellizzeri D., De Caro C., Calzetta L., Cuzzocrea S. (2020). Protective Effect of Hydroxytyrosol Against Oxidative Stress Induced by the Ochratoxin in Kidney Cells: In vitro and in vivo Study. Front. Veter- Sci..

[34] Gugliandolo E., D’Amico R., Cordaro M., Fusco R., Siracusa R., Crupi R., Impellizzeri D., Cuzzocrea S., Di Paola R. (2018). Effect of PEA-OXA on neuropathic pain and functional recovery after sciatic nerve crush. J. NeuroInflamm..

[35] Siracusa R., Paterniti I., Cordaro M., Crupi R., Bruschetta G., Campolo M., Cuzzocrea S., Esposito E. (2018). Neuroprotective Effects of Temsirolimus in Animal Models of Parkinson’s Disease. Mol. Neurobiol..

[36] Impellizzeri D., Siracusa R., Cordaro M., Crupi R., Peritore A.F., Gugliandolo E., D’Amico R., Petrosino S., Evangelista M., Di Paola R. (2019). N-Palmitoylethanolamine-oxazoline (PEA-OXA): A new therapeutic strategy to reduce neuroinflammation, oxidative stress associated to vascular dementia in an experimental model of repeated bilateral common carotid arteries occlusion. Neurobiol. Dis..

[37] Peritore A.F., Crupi R., Scuto M., Gugliandolo E., Siracusa R., Impellizzeri D., Cordaro M., D’Amico R., Fusco R., Di Paola R. (2020). The Role of Annexin A1 and Formyl Peptide Receptor 2/3 Signaling in Chronic Corticosterone-Induced Depression-Like behaviors and Impairment in Hippocampal-Dependent Memory. CNS Neurol. Disord. Drug Targets.

[38] Siracusa R., Impellizzeri D., Cordaro M., Crupi R., Esposito E., Petrosino S., Cuzzocrea S. (2017). Anti-Inflammatory and Neuroprotective Effects of Co-UltraPEALut in a Mouse Model of Vascular Dementia. Front. Neurol..

[39] D’Amico R., Cordaro M., Fusco R., Peritore A.F., Genovese T., Gugliandolo E., Crupi R., Mandalari G., Caccamo D., Cuzzocrea S. (2022). Consumption of Cashew (*Anacardium occidentale* L.) Nuts Counteracts Oxidative Stress and Tissue Inflammation in Mild Hyperhomocysteinemia in Rats. Nutrients.

[40] Cordaro M., Siracusa R., Fusco R., D’Amico R., Peritore A., Gugliandolo E., Genovese T., Scuto M., Crupi R., Mandalari G. (2020). Cashew (*Anacardium occidentale* L.) Nuts Counteract Oxidative Stress and Inflammation in an Acute Experimental Model of Carrageenan-Induced Paw Edema. Antioxidants.

[41] Fusco R., Cordaro M., Siracusa R., Peritore A.F., Gugliandolo E., Genovese T., D’Amico R., Crupi R., Smeriglio A., Mandalari G. (2020). Consumption of *Anacardium Occidentale* L. (Cashew Nuts) Inhibits Oxidative Stress through Modulation of the Nrf2/HO−1 and NF-kB Pathways. Molecules.

[42] Esposito E., Impellizzeri D., Bruschetta G., Cordaro M., Siracusa R., Gugliandolo E., Crupi R., Cuzzocrea S. (2016). A new co-micronized composite containing palmitoylethanolamide and polydatin shows superior oral efficacy compared to their association in a rat paw model of carrageenan-induced inflammation. Eur. J. Pharmacol..

[43] D’Amico R., Salinaro A.T., Fusco R., Cordaro M., Impellizzeri D., Scuto M., Ontario M., Dico G.L., Cuzzocrea S., Di Paola R. (2021). *Hericium erinaceus* and *Coriolus versicolor* Modulate Molecular and Biochemical Changes after Traumatic Brain Injury. Antioxidants.

[44] Impellizzeri D., D’Amico R., Fusco R., Genovese T., Peritore A.F., Gugliandolo E., Crupi R., Interdonato L., Di Paola D., Di Paola R. (2022). Açai Berry Mitigates Vascular Dementia-Induced Neuropathological Alterations Modulating Nrf-2/Beclin1 Pathways. Cells.

[45] Cordaro M., Fusco R., D’Amico R., Siracusa R., Peritore A.F., Gugliandolo E., Genovese T., Crupi R., Mandalari G., Cuzzocrea S. (2020). Cashew (*Anacardium occidentale* L.) Nuts Modulate the Nrf2 and NLRP3 Pathways in Pancreas and Lung after Induction of Acute Pancreatitis by Cerulein. Antioxidants.

[46] Ismail S., Rao K., Bhaskar M. (2016). Evaluation of anti-inflammatory activity of Boswellia serrata on carrageenan induced paw edema in albino Wistar rats. Int. J. Res. Med. Sci..

[47] Shimada K., Uzui H., Ueda T., Lee J.-D., Kishimoto C. (2015). N-Acetylcysteine Ameliorates Experimental Autoimmune Myocarditis in Rats via Nitric Oxide. J. Cardiovasc. Pharmacol. Ther..

[48] Kim K.-S., Hufnagel G., Chapman N., Tracy S. (2001). The group B coxsackieviruses and myocarditis. Rev. Med. Virol..

[49] Leuschner F., Katus H.A., Kaya Z. (2009). Autoimmune myocarditis: Past, present and future. J. Autoimmun..

[50] Zaki A.A., Hashish N.E., Amer M.A., Lahloub M.-F. (2014). Cardioprotective and antioxidant effects of oleogum resin “Olibanum” from Bos Boswellia carteri Birdw. (Bursearceae). Chin. J. Nat. Med..

[51] Ammon H.P.T. (2006). Boswellic Acids in Chronic Inflammatory Diseases. Planta Med..

[52] Shen T., Lou H.-X. (2008). Bioactive Constituents of Myrrh and Frankincense, Two Simultaneously Prescribed Gum Resins in Chinese Traditional Medicine. Chem. Biodivers..

[53] Forouzanfar F., Hosseinzadeh H., Bideskan A.E., Sadeghnia H.R. (2016). Aqueous and Ethanolic Extracts of *Boswellia serrata* Protect Against Focal Cerebral Ischemia and Reperfusion Injury in Rats. Phytother. Res..

[54] Ranjbarnejad T., Saidijam M., Moradkhani S., Najafi R. (2017). Methanolic extract of Boswellia serrata exhibits anti-cancer activities by targeting microsomal prostaglandin E synthase-1 in human colon cancer cells. Prostaglandins Other Lipid Mediat..

[55] Singh S., Khajuria A., Taneja S., Khajuria R., Singh J., Johri R., Qazi G. (2008). The gastric ulcer protective effect of boswellic acids, a leukotriene inhibitor from Boswellia serrata, in rats. Phytomedicine.

[56] Sharma R., Singh S., Singh G., Khajuria A., Sidiq T., Chashoo G., Pagoch S., Kaul A., Saxena A., Johri R. (2009). In vivo genotoxicity evaluation of a plant based antiarthritic and anticancer therapeutic agent Boswelic acids in rodents. Phytomedicine.

[57] Singh P., Chacko K.M., Aggarwal M.L., Bhat B., Khandal R.K., Sultana S., Kuruvilla B.T. (2012). A-90 day gavage safety assessment of Boswellia serrata in rats. Toxicol. Int..

[58] Sagar S., Liu P.P., Cooper L.T. (2012). Myocarditis. Lancet.

[59] Daniels M.D., Hyland K.V., Wang K., Engman D.M. (2008). Recombinant cardiac myosin fragment induces experimental autoimmune myocarditis via activation of Th1 and Th17 immunity. Autoimmunity.

[60] Li L.-Y., Wang X., Zhang T.-C., Liu Z.-J., Gao J.-Q. (2021). Cardioprotective effects of omega 3 fatty acids from fish oil and it enhances autoimmunity in porcine cardiac myosin-induced myocarditis in the rat model. Z. Nat. C.

[61] Afanasyeva M., Georgakopoulos D., Rose N.R. (2004). Autoimmune myocarditis: Cellular mediators of cardiac dysfunction. Autoimmun. Rev..

[62] Van der Borght K., Scott C.L., Nindl V., Bouché A., Martens L., Sichien D., Van Moorleghem J., Vanheerswynghels M., De Prijck S., Saeys Y. (2017). Myocardial Infarction Primes Autoreactive T Cells through Activation of Dendritic Cells. Cell Rep..

[63] Valaperti A., Marty R.R., Kania G., Germano D., Mauermann N., Dirnhofer S., Leimenstoll B., Blyszczuk P., Dong C., Mueller C. (2008). CD11b^+^ Monocytes Abrogate Th17 CD4^+^ T Cell-Mediated Experimental Autoimmune Myocarditis. J. Immunol..

[64] Kumar R., Singh S., Saksena A.K., Pal R., Jaiswal R., Kumar R. (2019). Effect of Boswellia serrata extract on acute inflammatory parameters and tumor necrosis factor-α in complete Freund’s adjuvant-induced animal model of rheumatoid arthritis. Int. J. Appl. Basic Med. Res..

[65] Tsutsui H., Kinugawa S., Matsushima S. (2008). Oxidative Stress and Mitochondrial DNA Damage in Heart Failure. Circ. J..

[66] Gomaa A.A., Mohamed H.S., Abd-Ellatief R.B., Gomaa M.A. (2021). Boswellic acids/Boswellia serrata extract as a potential COVID-19 therapeutic agent in the elderly. Inflammopharmacology.

[67] Barakat B.M., Ahmed H.I., Bahr H.I., Elbahaie A.M. (2018). Protective Effect of Boswellic Acids against Doxorubicin-Induced Hepatotoxicity: Impact on Nrf2/HO-1 Defense Pathway. Oxid. Med. Cell. Longev..

[68] Minj E., Upadhayay S., Mehan S. (2021). Nrf2/HO-1 Signaling Activator Acetyl-11-keto-beta Boswellic Acid (AKBA)-Mediated Neuroprotection in Methyl Mercury-Induced Experimental Model of ALS. Neurochem. Res..

[69] Chen M., Wang M., Yang Q., Wang M., Wang Z., Zhu Y., Zhang Y., Wang C., Jia Y., Li Y. (2016). Antioxidant effects of hydroxysafflor yellow A and acetyl-11-keto-β-boswellic acid in combination on isoproterenol-induced myocardial injury in rats. Int. J. Mol. Med..

[70] Di Paola D., Capparucci F., Lanteri G., Cordaro M., Crupi R., Siracusa R., D’Amico R., Fusco R., Impellizzeri D., Cuzzocrea S. (2021). Combined Toxicity of Xenobiotics Bisphenol A and Heavy Metals on Zebrafish Embryos (*Danio rerio*). Toxics.

